# Hantavirus RNA in Saliva from Patients with Hemorrhagic Fever with Renal Syndrome

**DOI:** 10.3201/eid1403.071242

**Published:** 2008-03

**Authors:** Lisa Pettersson, Jonas Klingström, Jonas Hardestam, Åke Lundkvist, Clas Ahlm, Magnus Evander

**Affiliations:** * Umeå University, Umeå, Sweden; † Swedish Institute for Infectious Disease Control, Solna, Sweden; ‡ Karolinska Institutet, Stockholm, Sweden

**Keywords:** Hantavirus, HFRS, HCPS, saliva, Puumala virus, transmission, zoonotic disease, research

## Abstract

Hantaviruses cause 2 zoonotic diseases, hemorrhagic fever with renal syndrome (HFRS) and hantavirus cardiopulmonary syndrome. Infection is usually initiated after inhalation of virus-contaminated rodent excreta. In addition to the zoonotic infection route, growing evidence suggests person-to-person transmission of Andes virus. For this reason, we studied whether saliva from HFRS patients contained hantavirus. During an outbreak in northern Sweden of nephropathia epidemica (NE), a milder form of hemorrhagic fever with renal syndrome, we collected saliva and plasma from 14 hospitalized NE patients with verified Puumala virus (PUUV) infection. PUUV RNA was detected in saliva from 10 patients (range 1,530–121,323 PUUV RNA copies/mL) by quantitative reverse transcription–PCR. The PUUV S-segment sequences from saliva and plasma of the same patients were identical. Our data show that hantavirus RNA could be detected in human saliva several days after onset of disease symptoms and raise the question whether interhuman transmission of hantavirus may occur through saliva.

## Abstract 2

Person-to-person transmission may occur through saliva.

Members of the family *Bunyaviridae* cause severe and often fatal human diseases in a large and increasing number of persons worldwide each year (*1*). This family contains 5 genera, and the genus *Hantavirus* causes 2 febrile illnesses: hemorrhagic fever with renal syndrome (HFRS) in Europe and Asia and hantavirus cardiopulmonary syndrome (HCPS) in North and South America. Hantaviruses are rodent-borne pathogens. In Sweden, Finland, Norway, Russia, and parts of central Europe, Puumala virus (PUUV) is endemic in bank voles (*Myodes glareolus*). PUUV causes nephropathia epidemica (NE), a milder form of HFRS. The most common symptoms of NE are fever, headache, nausea, vomiting, myalgia, abdominal pain, back pain, and visual disturbances (*2*). One third of the patients have hemorrhagic manifestations, 10%–20% have respiratory tract symptoms, and most have signs of renal failure (*2*). There is no effective treatment or available vaccine.

The most common route of hantavirus infection is infectious aerosols originating from saliva, urine, and feces of infected rodents (*1*). Because rodent bites have been demonstrated to cause human hantavirus infections (*3*,*4*), saliva of hantavirus-infected rodents must contain infectious virus. Infectious PUUV has been found in oropharyngeal secretions from *M. glareolus* (*5*) and Andes virus (ANDV) RNA is present in saliva from infected pigmy rice rats (*Oligoryzomys longicaudatus*) (*6*). Furthermore, Sin Nombre virus RNA is present in saliva but not in urine and feces from deer mice (*Peromyscus maniculatus*) (*7*), confirming that hantaviruses are transmitted to humans through rodent saliva. However, person-to-person transmission has recently been documented for the ANDV that causes HCPS (*8*–*13*), and contaminated human saliva suggests a possible route of infection (*13*). For herpesviruses, such as Epstein-Barr virus and herpesviruses-7 and -8, person-to-person transmission can occur from saliva (*14*–*16*), but human salivary secretions could also negatively modulate herpesvirus infectivity as shown for herpesvirus-1 (*17*).

Because the saliva of hantavirus-infected rodents contains infectious virus that is transmitted to humans (*3*,*4*,*7*) and person-to-person transmission is strongly suspected for ANDV (*13*), we studied whether the virus could be detected in saliva of hantavirus-infected patients. For this reason, we collected saliva from patients during an NE outbreak in northern Sweden and analyzed the samples for the presence and levels of PUUV RNA by using a real-time reverse transcription–PCR (RT-PCR) assay.

## Material and Methods

### Collection of Human Saliva

Saliva was collected from PUUV-infected patients who were hospitalized from January through May 2007 at the Department of Infectious Diseases at Umeå University Hospital (Umeå, Sweden). Samples were obtained from patients with typical clinical symptoms of acute NE, which were verified by detection of PUUV-specific immunoglobulin (Ig) M in serum, a PUUV-specific real-time RT-PCR, or both. The collection was random, with no consideration of time of the day or food intake. The samples were only collected on 1 occasion during each patient’s hospitalization by asking the patient to spit in a single-use plastic mug. The saliva was then removed with a syringe (5 mL; Becton Dickinson, Franklin Lakes, NJ, USA) and put in sterile plastic test tubes (Nunc, Roskilde, Denmark). The samples were immediately stored at –80°C until they were further analyzed. Plastic gloves and protective clothing were used during the procedure. A total of 9 samples were stored without additives, and 5 were diluted 1:3 in virus transport medium (2% HEPES, 10 μg/mL bovine serum albumin, 50 μg/mL sucrose, 0.016 μg/mL fungizone, and 2.5 μg/mL garamycin) before storage. The project was approved by the Research Ethics Committee of Umeå University and all patients gave written and informed consent.

### Real-Time RT-PCR

RNA from 140 μL of patient saliva and plasma was extracted by using a QIAamp Viral RNA kit (QIAGEN, Valencia, CA, USA) according to the manufacturer’s instructions. The real-time RT-PCR was performed as previously described (*18*). Briefly, the RNA was reverse-transcribed followed by a real-time PCR TaqMan assay in triplets with PUUV-specific primers and probe from the S segment (*18*). The real-time PCR was performed with an ABI Prism 7900HT Sequence Detection System 2.0 (Applied Biosystems, Foster City, California, USA). As internal positive control, we used a 72-bp *Drosophila melanogaster* fragment (nt position 1783–1854, GenBank accession no. NM_144343) cloned into a pcDNA3 vector (Invitrogen, Carlsbad, CA, USA). The plasmid was linearized with the restriction enzyme *Apa*I, and RNA was transcribed and purified as described (MEGAscript High Yield Transcription Kit, Ambion, Promega, Madison, WI, USA; www.megasoftware.net). The RNA control was diluted, added to patient sample, and used as template for quantitative real-time RT-PCR as described above. The primers and probe used for amplification of control RNA was *D. melanogaster* forward primer 5′-AGGTGCCCGTGTGTATCCAT-3′ (900 nM), reverse primer 5′-GCTCGTCCTCCGCCTCAT-3′ (900 nM) and probe 5′FAM-TACCACGAATCTGCGACATTACCAGGG-TAMRA-3′ (200 nM). Primers and probe were designed by using Primer Express version software 2.0 (Applied Biosystems).

### Immunofluorescence Assay

An immunofluoresence assay was performed as previously described (*18*). Briefly, patient serum was added to spot slides covered with fixed PUUV-infected Vero E6 cells, and IgG levels were determined by using fluorescein-conjugated rabbit anti-human IgG (F202, DAKO A/S, Glostrup, Denmark) diluted in phosphate-buffered saline (PBS) with Evans blue. For IgM analysis, patient serum was pretreated with rheumatoid factor absorbent (Virion\Serion GmbH, Würzburg, Germany) to eliminate possible interference of rheumatoid factor and PUUV-specific IgG. Slides were incubated overnight at 37°C, and antibody was detected by using fluorescein-conjugated rabbit F(ab′)2 anti-human IgM antibodies (F0317, DAKO A/S) diluted in PBS with Evans blue.

### Sequencing

cDNA from patient saliva and plasma were amplified by using Pfu DNA Polymerase (Fermentas Life Sciences, Helsingbord, Sweden), and the sequencing reactions were performed by using the Big Dye Terminator v 1.1 Cycle Sequencing kit (Applied Biosystems). The PCR and sequencing reactions were performed with primers from the S-segment. For patients 1 and 4, the primers for PCR was Puu1s (5′-CAAGAGGATATAACCCGCCA-3′) and Puu6as (5′-GCCATCCCTGCAACATAGAT-3′) followed by Puu3s (5′-AACTGGGATTGAGCCAGATG-3′) and Puu5as (5′-TGGGCATTCCTTTTCCATAA-3′) for sequencing. For patient 2, the primers for PCR was Puu1s and Puu5as followed by Puu2s (5′-ACCCGCCATGAACAACAACT-3′) and Puu4as (5′-TAGGGCTTTCAAAATAATAGGTAG-3′) for sequencing. Before the sequencing reaction, the PCR fragment was purified by using a QIAquick PCR Purification kit (QIAGEN) and precipitated in ethanol. Assembly, analysis, and alignment of sequences were performed by using Geneious Basic 3.0.6 (www.geneious.com) and BLAST (www.ncbi.nlm.nih.gov/blast).

## Results

### Hantavirus RNA in Saliva from NE Patients

We collected saliva 2–9 days after onset of symptoms from 14 hospitalized NE patients to determine whether hantaviruses were present in human saliva. All patients were positive for PUUV RNA in plasma by a real-time RT-PCR, and 13 patients had PUUV-specific IgM antibodies in serum (Table). We then analyzed the saliva by real-time RT-PCR for the presence and levels of viral RNA. Saliva samples from 10 of 14 patients were positive for PUUV RNA (range 1,530–121,323 PUUV RNA copies/mL), demonstrating that viral RNA could be detected in saliva during disease (Table). Furthermore, we detected no inhibition of real-time RT-PCR in saliva or plasma when we analyzed our patient samples by using an internal positive control (data not shown). All patients were also IgG positive in serum with varying titers.

**Table:** Comparison between detection of PUUV RNA in saliva and plasma and antibodies in serum samples from 14 patients with nephropathia epidemica*

Patient no.	Sex	Age, y	Date saliva collected (d of disease)	PUUV RNA† in saliva, copies/mL	PUUV RNA† in plasma,‡ copies/mL	PUUV IgM§ in serum‡	PUUV IgG titer in serum‡
1	M	65	6	121,323	959,294	+	40
2	M	64	2	66,994	117,562	+	80
3	M	33	5	44,898	40,427	+	>640
4	F	38	6	17,516	1,381,413	+	40
5	F	59	9	9,582	3,724	+	>640
6	M	54	5	6,372	26,626	–	320
7	M	39	7	3,745	189,233	+	320
8	M	28	5	2,589	36,231	+	>640
9	F	26	5	2,163	54,315	+	80
10	F	39	5	1,530	3,044	+	80
11	M	38	6	0	1,952	±	>640
12	M	41	6	0	5,215	+	40
13	F	57	2	0	41,271	+	320
14	F	62	7	0	81,330	+	160

*PUUV, Puumala virus; Ig, immunoglobulin. †Detected by real-time reverse transcription–PCR. ‡Most plasma and serum samples were collected the same day or within 1 day before or after the saliva sample and antibody was detected by immunofluorescence assay. §Serum was diluted 1:16 and scored as negative (–), positive (+), or weakly positive (±).

### Sequencing of PUUV RNA in Saliva

To study in more detail whether the RNA we detected in saliva was truly specific, we sequenced regions of RT-PCR products. Sequencing of amplification products from saliva and plasma from 3 NE patients—patient 1 (GenBank accession nos. EU337014 and EU337015), patient 2 (GenBank accession nos. EU177629 and EU177630), and patient 4 (GenBank accession nos. EU337016 and EU337017)—demonstrated a PUUV S-segment sequence that was identical for each saliva/plasma pair (data not shown). When we compared the sequences for patients 1 and 4, we found 24 mismatches in the 292-nt S-segment sequence, which clearly demonstrated that they were derived from different strains (Figure). The observed nucleotide mismatches did not result in different amino acid sequences. The sequence from patient 2 was from another S-segment region. All PUUV sequences from the NE patients were most closely related to PUUV strains from the disease-endemic region in northern Sweden and grouped with the northern branch of PUUV strains (*19*,*20*).

**Figure:**
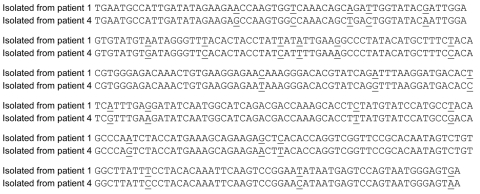
Comparison of nucleotide sequences between reverse transcription–PCR products isolated from nephropathia epidemica patients 1 and 4. Mismatches are underlined.

### Respiratory Symptoms in NE patients

All 14 NE patients required hospital care and displayed the symptoms of NE. All survived and none had to be treated by dialysis. Seven patients had respiratory symptoms including cough, dyspnea, or both. In 2 of the patients with respiratory symptoms, a chest radiograph revealed infiltrates (data not shown). The 2 patients with the highest levels of viral RNA in saliva had dyspnea (patient 1) and cough and lung infiltrates (patient 2) (Table). All patients with respiratory symptoms, except 1 (patient 12), had PUUV RNA in their saliva.

## Discussion

This investigation showed that hantavirus RNA could be detected in saliva from HFRS patients. We found PUUV RNA with varying PUUV genome copy numbers in the saliva of most of the 14 hospitalized NE patients. Previously, we have shown that PUUV RNA in plasma decreases with time in the individual patient (*18*), and although we did not study this finding, PUUV in saliva likely would display similar kinetics. PUUV viremia is thought to persist for 5–7 days, but in our study, 1 patient had PUUV in both saliva and plasma 9 days after onset of symptoms. Furthermore, we recently detected PUUV RNA in plasma 16 days after first appearance of disease (*18*). It would also be interesting to study whether nonhospitalized patients with milder symptoms also had PUUV RNA in their saliva. We do not know if PUUV RNA in saliva could originate from PUUV viremia, but the PUUV RNA detected in saliva was likely produced in salivary glands or through coughing. Recently, ANDV antigen was found in secretory cells of the salivary glands of human patients (*21*). In our study, 7 of the patients had respiratory symptoms, and virus shedding in saliva is known to occur for several respiratory viruses, such as severe acute respiratory syndrome–associated coronavirus, respiratory syncytial virus, and human metapneumovirus (*22*,*23*). Symptoms in the upper respiratory tract are also often present in NE (*2*,*24*), which makes our finding of PUUV in saliva credible. One may argue that NE, the HFRS endemic in northern and central Europe, is a different entity than HCPS, caused by Sin Nombre hantavirus and ANDV, and that the finding of viral RNA in saliva from NE patients is a pure coincidence. However, NE in Scandinavia shows several common characteristics with HCPS in the Americas. For example, pulmonary involvement and respiratory symptoms are common in NE. In addition, radiologic examination–detectable infiltrates, decreased pulmonary function, and a local inflammatory response in the lungs have previously been demonstrated (*25*–*28*).

Could hantaviruses in human saliva be infectious and initiate infection? Today, the only hantavirus suggested to be transmitted between humans is ANDV (*8*–*13*). By using sophisticated epidemiologic data, Ferres et al. showed that the risk for HCPS caused by ANDV was higher among sex partners of the index case than among other household contacts (*13*). These investigators suggested that ANDV needs close person-to-person contact, such as sexual relations or deep kissing, to be transmitted between humans (*13*). Our finding of hantavirus in saliva support the conclusion that hantavirus infection could be transmitted between humans through saliva, such as during kissing or coughing. However, we do not know at this stage whether the hantavirus RNA in saliva detected in our study is infectious. When we tried to infect bank voles with human PUUV RNA–positive saliva, no seroconversion was found after 21 days (J. Hardestam et al., unpub. data). Furthermore, we have not been successful in isolating PUUV from saliva specimens by infecting Vero E6 cells. Clinical isolates of hantaviruses do not grow readily on cell lines, and mutations of the noncoding regions were shown to be needed for the PUUV strain Kazan, originally isolated from bank voles, to grow in Vero E6 cells (*29*). The only human PUUV isolate from Sweden, PUUV strain Umeå/hu, adapted to growth in Vero E6 cells, was isolated with phytohemagglutinin-stimulated leukocytes from an NE patient, and PUUV antigen was not detected until 6 months after infection of Vero E6 cells (*30*).

How long after onset of disease viral hantavirus RNA is present in saliva and whether saliva contains neutralizing antibodies need to be studied further, even though ANDV-specific IgA antibodies have been detected in saliva in patients with acute HCPS from 5 to 31 days after onset of symptoms (*31*). Detecting hantavirus-specific antibodies in human saliva may be useful for diagnostics, and detecting viral RNA in saliva could be used as a tool to study hantavirus epidemiology. ANDV RNA was shown to be present in human peripheral blood cells for 5 to 15 days before the onset of symptoms (*13*), and PUUV RNA was detected in plasma from *Cynomolgus* macaques 4 days before symptoms appeared (*32*). Studies are needed to determine whether the same is true for saliva in animal models. If this was the case, the hantavirus in antibody-free saliva could have a rather high infectious potential. On the other hand, saliva has antimicrobial functions (*33*) and has been shown to inhibit certain viruses, such as HIV-1 (*34*), influenza A virus (*35*), and herpes simplex virus type 1 (*17*); however, saliva does not inhibit Epstein-Barr virus (*14*) and adenovirus (*34*). So far, little is known regarding the effect of human saliva on hantavirus infectivity, but results of experiments we have performed indicate that human saliva could reduce at least part of the infectivity in vitro (J. Hardestam et al., unpub. data). Apparently, hantaviruses in rodent saliva are infectious, but whether the composition of rodent and human saliva differs is not known. Further studies are needed to elucidate the mechanisms behind hantavirus transmission between the natural rodent hosts and humans.

After finding PUUV RNA in saliva from NE patients, we searched for evidence of possible person-to-person transmission of PUUV. When the NE patients were followed-up 1–2 months later, we discovered several clusters of household members with NE symptoms. However, none had severe enough disease to require physician care, and no samples were collected for laboratory diagnosis. Furthermore, during the large NE outbreak in early 2007, there was a very high incidence (313/100,000) of NE in the region where our university hospital is situated (*36*). Since all patients lived in this area, we cannot exclude inhalation of rodent excreta as the main or only transmission route. Although it remains to be clearly shown, our results support the hypothesis that person-to-person transmission may occur by this route.
